# Photobiomodulation Therapy in the Treatment of Oral Mucositis, Dysgeusia and Oral Dryness as Side-Effects of Head and Neck Radiotherapy in a Cancer Patient: A Case Report

**DOI:** 10.3390/dj6040064

**Published:** 2018-11-10

**Authors:** Marwan El Mobadder, Fadi Farhat, Wassim El Mobadder, Samir Nammour

**Affiliations:** 1 Department of Dental Science, Faculty of Medicine, University of Liège, 4000 Liège, Belgium; S.Namour@ulg.ac.be; 2 Department of Hematology and Oncology, Hammoud Hospital University Medical Centre, 652 Saida, Lebanon; drfadi.research@gmail.com; 3 Department of Endodontics, Faculty of dental medicine, University Saint Joseph, Beirut 1107 2050, Lebanon; drmobader@gmail.com

**Keywords:** photobiomodulation, low-level laser therapy, dysgeusia, oral mucositis, oral dryness, hyposalivation, cancer

## Abstract

Successful management of oral mucositis, dysgeusia and oral dryness was made with five sessions of photobiomodulation. The severity of oral mucositis was measured according to the World Health Organization scale for the assessment of oral mucositis. Dysgeusia testing was performed according to the International Standards Organization (ISO). For the assessment of oral dryness or hyposalivation, quantity of the total resting and stimulated saliva (Q-sal, mL/min) was measured. Photobiomodulation parameters, applications, and treatment protocol used were suggested by an international multidisciplinary panel of clinicians and researchers with expertise in the area of supportive care in cancer and/or PBM clinical application and dosimetry. This case report confirms the effectiveness of photobiomodulation therapy in the management of oral mucositis, dysgeusia, and oral dryness.

## 1. Introduction

Photobiomodulation (PBM) therapy is defined as a form of light therapy. Visible, infrared and near infrared light is absorbed by endogenous chromophores, triggering biological reactions that are not thermal or cytotoxic through photochemical or photophysical events, leading to physiological changes. It is now established that radiation at certain wavelengths can modulate living cells and tissues [1]. Photobiomodulation was previously known as low-level laser therapy because the light used is of low intensity compared to other forms of medical laser treatments, which are mainly used for surgery [1]. Recent data suggests that the mechanism of action of PBM is predominantly related to an action on cytochrome c oxidase (CcO) in the mitochondrial respiratory chain by facilitating electron transport [2,3]. This, therefore, results in an increased transmembrane proton gradient, which drives adenosine triphosphate (ATP) production, and in an increased bioavailability to power the functions of cellular metabolism [2,3]. PBM enhances wound repair and tissue regeneration by acting on different phases of injury resolution, including inflammation, proliferation, and remodeling phases [4].

Chemotherapy (CT) and/or radiotherapy (RT) are effective treatment modalities for cancer patients. Nevertheless, these treatments are associated with side-effects such as mucositis, dysphagia, dysgeusia, radiation dermatitis, xerostomia or hyposalivation, and head and neck lymphedema, among others [5]. In fact, 80% of patients receiving a high dose of CT prior to hematopoietic stem cell transplantation (HSCT) manifest oral mucositis. Patients receiving conventional CT for solid tumors have a 20–40% risk of manifesting oral mucositis, whereas patients receiving head and neck RT have an 80% risk of the same [6]. 63–93% of patients exhibit xerostomia or hyposalivation when the zone of radiation includes the salivary glands [7]. Dysgeusiais was found in approximately 66.5% of patients receiving head and neck RT alone and in 76.0% of patients after they have undertaken head and neck chimioradiotherapy (CRT) [8]. Approximately 15% of patients continue to experience dysgeusia after treatment [8].

The use of PBM has recently shown significant promise for supportive cancer care measures [9]. In fact, the Multinational Association of Supportive Care in Cancer and the International Society of Oral Oncology recommend the use of photobiomodulation for the prevention of oral mucositis in two cases: for patients having head and neck cancer and for patients before undergoing hematopoietic stem cell transplant [10,11]. On the other hand, an international multidisciplinary panel of clinicians and researchers with expertise in the area of supportive care in cancer and/or PBM clinical application and dosimetry have recently suggested treatment protocols in the management of side effects due to radio and/or chemotherapy. Different protocols have been proposed for the prevention and treatment of the following complications: oral mucositis, dysphagia, dysgeusia, hyposalivation or xerostomia, Radiation dermatitis, trismus, osteonecrosis, head and neck lymphedema and voice speech alteration due to local inflammation [8,9].

## 2. Case Report

A 48-year-old male patient diagnosed with adenocarcinoma consistent with salivary duct carcinoma T4a (40 mm) N2 (multiple focal 1 cm right submandibular and retrocervical nodes) M0/stage IVA. Surgical excision was not recommended; the patient underwent Intensity-ModulatedRadiation Therapy with a curative aim for 44 days. After radiotherapy, the patient started to complain of pain, dysgeusia, and oral dryness, which persisted with time. Oral mucositis, dysgeusia, and oral dryness were diagnosed based on a meticulous clinical examination. No ethical committee approval was necessary for our research since the protocol used in this case report is well described in the literature. The patient signed a written informed consent before enrolling in the study.

Assessment of oral mucositis

The severity of oral mucositis was measured according to the World Health Organization scale for assessing oral mucositis. The patient presented erythema and ulcers but was able to eat solid foods; therefore, the severity of oral mucositis was Grade 2 (Table 1).

Assessment of dysgeusia

According to the International Standards Organization (ISO), dysgeusia test was used in order to assess the severity of the disorder. Before all taste tests, the patient was asked to stop eating, drinking, smoking or using any oral care products. The patient was also required to drink only water at least one hour prior to testing. Five 2-mm solutions that represent basic taste qualities—sweet, salty, sour, bitter and umami—were tasted in a single ‘sip and spit’ technique. The solutions and their corresponding concentrations were sucrose 300 mM, NaCl 200 mM, citric acid 5 mM, caffeine 10 mM, and MSG 200 mM. Perceived taste quality was identified by selecting one of seven choices. Correct responses were sweet for sucrose, salty for NaCl, sour for citric acid, bitter for caffeine, and savory for MSG. Further choices were none or metallic. Taste identification score was assigned as 0–5 correct choices. The test identification score was 3 out of 5. The mouth was rinsed with purified water 3 times before and after sampling and expectorating each solution (Table 1).

Assessment of oral dryness

For the assessment of oral dryness or hyposalivation, the quantity of the total resting and stimulated saliva (Q-sal, mL/min) was measured. The patient was asked to expectorate all saliva into a graduated test tube for a 10-min period before the citric acid stimulation and a 5-min period after the stimulation. The amount of saliva before and after stimulation was determined by the scale on the graduated tube. The quantity of saliva was respectively 0.05 and 0.11 for the resting and stimulating saliva [12]. After the assessment of the severity of oral mucositis, dysgeusia, and oral dryness, the treatment of choice was the therapeutic (curative) use of photobiomodulation (low-level laser therapy) for at least 5 times a week until there was an improvement in symptoms. Photobiomodulation dosimetry, applications, and treatment protocol was based on evidence derived from the literature and expert opinion that provided a guideline for the use of photobiomodulation in supportive cancer care for oral mucositis, dysgeusia and oral dryness [9] (Table 1).

i. Treatment of oral mucositis

Intraoral application of diode laser is employed with a wavelength of 980 nm (FONA Laser Sirona Dental Systems GmbH, Germany) at energy density of 4 J/point and a time of 12 s per point in a continuous and contact mode on 4 points on the tongue and 2 on the oropharynx. Extraoral application of diode laser is achieved with a wavelength of 980 nm (FONA Laser Sirona Dental Systems GmbH, Germany), energy density of 4 J/point, and a time of 12 s per point on the following areas: lips, cutaneous surface corresponding to the buccal mucosae, and bilateral cervical lymphaphatic chain [9] (Table 2).

ii. Treatment of dysgeusia

Intraoral application of diode laser is achieved with a wavelength of 980 nm (FONA Laser Sirona Dental Systems, GmbH, Germany) at 10 points on the dorsal and lateral tongue at an energy density of 3 J/cm^2^ for 12 s on each point. Each session was repeated 3 times for one week, within 48 h between each session [9] (Table 2).

iii. Treatment of Oral dryness/hyposalivation

Extraoral application of diode laser 650 nm (LLLT Laser Bio:PDT: Soulagement de douleur par MG Consiliis) is attained with a total of 20 points (10 each side) at an energy density of 3 J/cm^2^ for 12 s in a continuous and contact mode targeting major salivary glands (parotid and submandibular glands) and minor salivary glands in each side [9].

Five sessions of photobiomodulation were implemented for one week as follows: Monday Tuesday, Wednesday, Thursday, and Friday. After 24 h of each session, assessments of oral mucositis, oral dryness and dysgeusia were made according to the already mentioned scores and criteria. After each session, the severity of oral mucositis and oral dryness decreased, and taste perception improved. Oral mucositis decreased from grade 2 to grade 0 after five sessions of PBM. The patient scored 5 out of 5 on the taste identification score. The quantity of whole resting and stimulated saliva increased from 0.05 to 0.12 for the resting saliva and from 0.11 to 0.21 for the stimulated saliva. Table 1 shows the results.

## 4. Discussion

Oral complications induced by radiotherapy and/or chemotherapy represent an important clinical challenge, which affects the quality of life. These complications might delay cancer therapy or compromise with the adherence of patients to the treatment [13]. The most common oral and neck complications of cancer therapy are oral mucositis, dysphagia, dysgeusia, hyposalivation or xerostomia, osteonecrosis, radiation dermatitis, head and neck lymphedema, and trismus [8]. In this case report, the patient suffered from oral mucositis, dysgeusia and oral dryness. According to the National Cancer Institute (NCI), oral mucositis is defined as the inflammation of the mucous membrane, which lines the digestive tract from the mouth to the anus. Multiple mechanisms can lead to mucositis, including DNA damage, production of the reactive oxygen species and epithelial atrophy, bacterial translocation, and profound inflammation [14]. Eighty percent of patients receiving head and neck radiotherapy exhibit oral mucositis [9]. Currently, cohrane meta-analysis [15], systematic review and meta-analysis [16,17], and other high-quality studies support the efficacy of photoboimodulation in the management of oral mucositis for patients undergoing chemotherapy or chemoradiotherapy. However, the authors agree that it is essential to recognize optimal PBM parameters per cancer treatment modality according to the severity of the symptoms of each patient. The Multinational Association of Supportive Care in Cancer and the International Society of Oral Oncology recommends the preventive use of photobiomodulation for patients receiving head and neck radiotherapy [11]. Another important complication of RT to the head and neck region is hyposalivation or oral dryness. In fact, 63–93% of patients show xerostomia or hyposalivation when the zone of radiation includes the salivary glands. Approximately all patients suffered from xerostomia as a result of radiation in the head and neck area due to RT [12]. A reduction in salivary glands’ function has an important impact on the patients’ quality of life and an increased burden of oral, dental care, and nutrition on a long term [18]. In contrast to oral mucositis, the literature on photobiomodulation for the management of hyposalivation is still limited. Therefore, this clinical report points out the potential use of PBM in the management of oral dryness or hyposalivation. Dysgeusia, commonly known as taste alteration during cancer therapy, is not well perceived in the literature; however, CT and RT cause dysgeusia by destroying rapidly dividing taste bud cells and olfactory receptor cells [19]. In this report, the patient underwent head and neck radiotherapy; therefore, oral mucositis, oral dryness and dysgeusia are expected symptoms. As a result, the patient’s oral mucositis was treated, salivation and salivary function was significantly better, and taste perception was restored. This case report shows that photobiomodulation can effectively and safely reduce the severity of oral mucositis, oral dryness, and dysgeusia in only five sessions. Photobiomodulation can be considered as an effective and relatively fast approach to enhance the quality of life of cancer patients that present severe oral complications. It is important to note that these positive results were obtained using the parameters, applications, and treatment protocol suggested by international multidisciplinary board of researchers and experts in the area of photobiomodulation and supportive cancer care. As already noted, literature on the use of PBM in the management of dysgeusia and oral dryness is limited. Therefore, this study has contributed to making efficient progress in the research process where positive results were shown, so as to back previous literature. It is important to note that investigations are being made to see if PBM itself poses a carcinogenic threat or has the potential to alter (proliferate or invade) established tumor behaviors [20]. However, in this report, the risk was not there, because the application of PBM was made after cancer therapy.

## 5. Conclusions

Within the limitation of this clinical report, it can be concluded that the therapeutic use of photobiomodulation according to specific protocol is considered a good approach for the treatment of oral mucositis, dysgeusia, and oral dryness after head and neck radiotherapy.

## Figures and Tables

**Table 1:** Results of the assessments of oral mucositis, dysgeusia, and oral dryness.

Complication	T Initial	T1	T2	T3	T4	T5
Oral mucositis score according to the WHO	2	2	1	1	0	0
Dysgeusia	3/5	3/5	4/5	5/5	5/5	5/5
Quantity of whole resting and stimulated saliva (Q-sal, mL/min):						
Before stimulation	0.05	0.06	0.06	0.07	0.1	0.12
After stimulation	0.11	0.15	0.16	0.18	0.25	0.27

T initial = before treatment, T1 = 48 h after first session, T2 = 48 h after the second session. T3 = 48 h after the third session. T4 = 48 h after the fourth session. T5 = 48 h after the fifth session. Oral mucositis score according to WHO, dysgeusia measurements according to ISO taste identification score. Quantity of whole resting and stimulated saliva in mL per minute (mL/min).

**Table 2:** Photobiomodulation parameters: applications and treatment protocol.

Complication	Parameters	ZoneIrradiated
Oral mucositis	Intraoral application: Diode laser 980 nm, energy density of 4 J/point and a time of 12 s per point in a continuous and contact mode Extraoral application: Diode laser 980 nm, energy density of 4 J/point, 12 s per point	Intraoral application: 4 points on the tongue and 2 on the oropharynx. Extraoral application: Lips, cutaneous surface corresponding to the buccal mucosae, bilateral cervical lymphatic chain.
Dysgeusia	Intraoral application Diode laser 980 nm. 10 points. Energy density of 3 J/cm^2^ for 12 s on each point.	Intraoral application Dorsal and lateral tongue.
Oral dryness	Extraoral application Diode laser 650 nm. Energy density of 3 J/cm^2^ for 12 s. In continuous and contact mode. A total of 10 points (1 cm per point)	Extraoral application Major salivary glands: parotid and submandibular glands. Minor salivary glands in each side.
